# MicroRNAs as Potential Biomarkers of Environmental Exposure to Polycyclic Aromatic Hydrocarbons and Their Link with Inflammation and Lung Cancer

**DOI:** 10.3390/ijms242316984

**Published:** 2023-11-30

**Authors:** Pablo Letelier, Rolando Saldías, Pía Loren, Ismael Riquelme, Neftalí Guzmán

**Affiliations:** 1 Laboratorio de Investigación en Salud de Precisión, Departamento de Procesos Diagnósticos y Evaluación, Facultad de Ciencias de la Salud, Universidad Católica de Temuco, Temuco 4813302, Chile; ulises.saldias.roa@gmail.com (R.S.); nguzman@uct.cl (N.G.); 2 Center of Molecular Biology and Pharmacogenetics, Scientific and Technological Bioresource Nucleus, Universidad de La Frontera, Temuco 4811230, Chile; pia.loren@ufrontera.cl; 3 Institute of Biomedical Sciences, Faculty of Health Sciences, Universidad Autónoma de Chile, Temuco 4810101, Chile; ismael.riquelme@uautonoma.cl

**Keywords:** polycyclic aromatic hydrocarbons, pulmonary cancer, lung cancer, microRNAs, particulate matter

## Abstract

Exposure to atmospheric air pollution containing volatile organic compounds such as polycyclic aromatic hydrocarbons (PAHs) has been shown to be a risk factor in the induction of lung inflammation and the initiation and progression of lung cancer. MicroRNAs (miRNAs) are small single-stranded non-coding RNA molecules of ~20–22 nucleotides that regulate different physiological processes, and their altered expression is implicated in various pathophysiological conditions. Recent studies have shown that the regulation of gene expression of miRNAs can be affected in diseases associated with outdoor air pollution, meaning they could also be useful as biomarkers of exposure to environmental pollution. In this article, we review the published evidence on miRNAs in relation to exposure to PAH pollution and discuss the possible mechanisms that may link these compounds with the expression of miRNAs.

## 1. Introduction

Since 1970, global concern has grown about the effect of environmental pollution on health [1,2], whereby epidemiological studies have shown a significant association between increased mortality and exposure to certain pollutants in outdoor air [3,4]. Among the contaminants present in the air, a combination of particles defined as particulate matter (PM) has been described, whose sustained exposure over time is associated with cardiovascular and respiratory diseases [5,6,7]. According to the World Health Organization (WHO), more than 50% of PM_2.5_ pollution worldwide is emitted by countries located in Africa, Europe and the Eastern Mediterranean [8].

The pathophysiological effects produced by PM are mainly due to the size of the particles and their complex composition which may include volatile metals, inorganic ions, volatile organic compounds (VOCs), and their derivatives [5,9,10]. Polycyclic aromatic hydrocarbons (PAHs) are a class of organic compounds that have two or more structural benzene rings, which are emitted into the environment by incomplete combustion of various organic components, such as fossil fuels or biomass. Depending on the weather conditions, emission sources and properties of the PAHs, they can be found in the gas phase or be part of the PM [11]. Various studies have correlated PM PAHs with respiratory system diseases such as asthma, chronic bronchitis, pulmonary emphysema, chronic inflammation, and lung cancer [12,13,14,15,16,17].

The respiratory tract is constantly in contact with various pollutants, so PAHs tend to concentrate in bronchial epithelial cells [18]. Due to their lipophilic nature, they can easily penetrate the epithelial barrier and accumulate in adipocytes, which leads to chronic activation of the aryl hydrocarbon receptor (AhR) [19], triggering an inflammatory response. Furthermore, PAH molecules that enter lung cells are considered pro-carcinogenic because although they do not directly induce DNA damage, they activate metabolic processes driven by cytochrome P450 enzymes, leading to the formation of oxidized PAHs that can promote DNA mutagenesis [20]. The mechanisms described to date have correlated the ability of environmental exposure to generate mutations in DNA; however, evidence also suggests that these environmental factors can cause epigenetic changes that can increase the risk of disease. In particular, environmental factors have been related to the altered expression of microRNAs (miRNAs or miRs) [21,22].

MiRNAs correspond to small non-coding RNA sequences (~22 nucleotides) that regulate gene expression [23] and are involved in the regulation of a wide variety of biological processes [24]. Their expression has been found to be deregulated in practically all human cancers [25], inflammatory diseases [26], and respiratory system pathologies, among other abnormalities [27]. Currently, multiple studies have linked the deregulation of their expression with environmental chemicals and related diseases [28,29,30].

In this article, we review the available evidence on miRNAs whose expression patterns are related to exposure to environmental PAHs. Also, we discuss the possible mechanisms by which these compounds may induce alterations of miRNA expression and the pathophysiology of respiratory diseases related to exposure to PM and PAHs.

## 2. Composition of Particulate Matter

Solid particles and liquid droplets can be found in suspension in breathed air, forming a matrix that contains a wide range of chemical species. This matrix is termed PM and includes inorganic acids, volatile metals, and a complex mixture of VOCs and volatile organic elements, whose characterization commonly reveals the presence of PAHs (e.g., Phenanthrene, Fluoranthene, Pyrene, Benzopyrene) [10,31] in addition to benzene, formaldehyde, and acetaldehyde. These, together with diesel engine emissions (DEP) and wood smoke particles (WSP) make up the VOCs present in urban air, all in variable concentrations [5,32,33]. PM is a key indicator of air pollution, being a factor associated with an increase in hospital admissions, exacerbation of respiratory disease, lung cancer and premature mortality, among other indicators [34,35,36]. According to International Agency for Research on Cancer (IARC) reports, it has been noted that exposure to PM promotes a state of oxidative stress and inflammation that contributes to the onset and progression of cancer [15].

PMs can be classified according to their aerodynamic diameter. Particles smaller than 10 microns (PM_10_) are retained in upper areas of the respiratory system and are removed by mucociliary action and mechanical processes [1,9]. Interestingly, there is also a finer fraction that represents more than 50% of the total PM composition and whose diameter spans 2.5 to 0.1 microns, called PM_2.5_ [9,37]. This PM2.5 has been shown to contain higher amounts of metals and PAHs and can reach terminal bronchioles and alveoli to potentially promote lung inflammation, to finally also access systemic circulation due to its small size [9,37,38]. Therefore, as the aerodynamic size of the particle decreases, the transport and penetration capacity into the respiratory system rises, contributing to greater pulmonary toxicity [9]. For this reason, the exposure for at least 3 years to PM_2.5_ is associated with a greater incidence of lung cancer due to an increase in mutation of *EGFR* and *KRAS* [39].

### Polycyclic Aromatic Hydrocarbons in Particulate Matter

PAHs make up an important part of the PM matrix, and their presence in the environment is mainly due to the incomplete combustion of fossil fuels. Emission sources are mainly industrial, vehicular, commercial and residential [11,40]. A multicenter study that evaluated emissions and sources of PAHs between 1960 and 2008 revealed that domestic burning of biomass makes up 60.5% of total emissions, and constitutes a major source of high-molecular-weight PAHs [11,41]. 

PAHs can be divided into two groups according to the number of aromatic rings. Specifically, low-molecular-weight PAHs are those made up of fewer than four rings, whilst high-molecular-weight PAHs are composed of four or more rings (e.g., Benzo[a]pyrene, Diben-zo[a,h]anthracene) [11]. Additionally, PAHs are highly lipophilic and their physiological action is dependent on their structure and metabolization [11]. Furthermore, they can be photo-oxidized and degraded to more reactive substances that result in newly formed compounds, such as oxidative and hydroxylated metabolites (OH-HAPs) and reactive epoxides [42]. Thus PAHs have been the subject of numerous studies that highlight the risks and adverse effects on human health (Table 1) [11,43]. 

It has been described that their harmful effects depend mainly on the degree of exposure, concentration, toxicity and route of exposure [11]. Regarding the exposure route, PAHs may enter humans by respiration, ingestion through the diet, dermal absorption, breastfeeding and placental transfer. Additionally, some exposures may involve more than one route simultaneously, a condition that affects the total absorbed dose [44]. PAHs are metabolized mainly in the liver and lungs, and can be excreted in breast milk and stored in adipose tissue [44,45]. Metabolization is complex and is specific for each metabolite. For example, Ben-zo[a]pyrene (B[a]P) passes into systemic circulation through type I epithelial cells in the alveolar region, and is subsequently transformed into reactive epoxides, before being metabolized in the liver by the enzyme complex (CYP P-450), for hepato-biliary excretion and elimination through feces and urine [46,47,48]. In addition, PAHs can be metabolized in the adrenal gland, testicles, thyroid, lungs, skin, and sebaceous glands [47].

**Table 1:** List of carcinogenic polycyclic aromatic hydrocarbons (PAHs) according to classification by The International Agency for Research on Cancer (IARC).

Name	Molecular Weight	Molecular Formula	Cas. No.	IARC Carcinogenic [49]
Anthracene	178.23 g/mol	C_14_H_10_	120–12–7	Group 2B
Anthraquinone	208.21 g/mol	C_14_H_8_O_2_	84–65–1	Group 2B
Benz[a]anthracene	228.3 g/mol	C_18_H_12_	56–55–3	Group 2B
Benzo[b]fluoranthene	252.3 g/mol	C_20_H_12_	205–99–2	Group 2B
7,8-Benzfluoranthene	252.3 g/mol	C_20_H_12_	205–82–3	Group 2B
Benzo[k]fluoranthene	252.3 g/mol	C_20_H_12_	207–08–9	Group 2B
Benzo[c]phenanthrene	228.3 g/mol	C_18_H_12_	195–19–7	Group 2B
Benzo[a]pyrene	252.3 g/mol	C_20_H_12_	50–32–8	Group 1
Carbazole	167.21 g/mol	C_12_H_9_N	86–74–8	Group 2B
Chrysene	228.3 g/mol	C_18_H_12_	218–01–9	Group 2B
Cyclopenta[cd]pyrene	226.3 g/mol	C_18_H_10_	27208–37–3	Group 2A
Dibenz[a,h]acridine	279.3 g/mol	C_21_H_13_N	226–36–8	Group 2B
Dibenz[a,j]acridine	279.3 g/mol	C_21_H_13_N	224–42–0	Group 2A
Dibenz[c,h]acridine	279.3 g/mol	C_21_H_13_N	224–53–3	Group 2B
Dibenz[a,h]anthracene	278.3 g/mol	C_22_H_14_	53–70–3	Group 2A
7H-Dibenzo[c,g]carbazole	267.3 g/mol	C_20_H_13_N	194–59–2	Group 2B
Dibenzo[b,def]chrysene	302.4 g/mol	C_24_H_14_	189–64–0	Group 2B
Dibenzo[a,i]pyrene	302.4 g/mol	C_24_H_14_	189–55–9	Group 2B
Dibenzo[a,l]pyrene	302.4 g/mol	C_24_H_14_	191–30–0	Group 2A
Indeno[1,2,3-cd]pyrene	276.3 g/mol	C_22_H_12_	193–39–5	Group 2B
Naphthalene	128.17 g/mol	C_10_H_8_	91–20–3	Group 2B
Quinoline	129.16 g/mol	C_9_H_7_N	91–22–5	Group 2B

Standard IARC classification: Group 1: “Carcinogenic to humans”; Group 2A: “Probably carcinogenic to humans with strong evidence”; Group 2B: “Possibly carcinogenic to humans with some evidence”. PAHs classified as Group 3 were not included in this table because they are not listed as carcinogenic to humans.

## 3. Pathophysiology of Respiratory Disease Related to Exposure to PM and PAHs

The WHO has estimated that around a quarter of the diseases that affect humans result from prolonged exposure to environmental pollution [50]. The air quality model presented in 2016 indicates that 92% of the world’s population lives in places where pollution levels exceed the limits set by the WHO [51]. Additionally, more than two million premature deaths each year can be attributed to the toxic effects of air pollution [52]. The five respiratory conditions with the greatest impact on people’s health are chronic obstructive pulmonary disease (COPD), asthma, acute respiratory infections, tuberculosis and lung cancer [53], this last pathology being the one with the worst prognosis [54,55]. Various studies have linked lung cancer with exposure to PAHs and DEPs present in urban air, demonstrating their carcinogenic effects on the lung [56] and close linkage to inflammation of the respiratory system [57,58].

PM acts as an important inducer of pro-inflammatory mediators [59,60]. It has been reported that VOCs induce an increase in cytokines and pro-inflammatory mediators in human bronchial epithelial cells (BEAS-2B) [10,61], a finding that correlates with an increase in inflammatory mediators (e.g., TNF-α, IL-6 and IL-8) in alveolar macrophages and type I pneumocytes in response to WSP [62]. In BEAS-2B cells, exposure to DEP present in PM generates greater susceptibility to airway inflammation and decreased lung function [63,64] and an increase in the expression of cyclooxygenase type 2 (COX-2) and the transcription factor STAT3 [61,65], in addition to the activation of the mitogen-activated protein kinase pathway (MAPK) that results in the activation of the NF-Kβ factor in human aortic endothelial cells (HAEC) [66,67]. Studies of exposure to WSP and DEP indicate deregulation in the expression of genes related to inflammation, DNA repair and apoptosis in lung cells [68,69,70]. Additionally, a study that evaluated 2360 participants over 20 years of age found a positive association between PM exposure and ultra-sensitive C-reactive protein (hs-CRP) levels [71]. Although PM_2.5_ air pollution has gained much attention in recent years as a factor associated with the increase in the incidence of respiratory diseases [72], the current vision has also focused on describing the factors related to the inflammatory response and carcinogenesis of lung cancer [72,73].

One of the mechanisms involved in the initial damage to the pulmonary system due to the induction of PAHs is related to the generation of reactive oxygen species (ROS), oxidative stress and inflammation [73,74]. Accordingly, three main mechanisms have been described to explain this phenomenon: (a) direct action through the formation of reactive metabolites (e.g., Ben-zo[a]pyrenediolepoxide) produced by the metabolization of PAHs by the CYP-complex P450, generating a high rate of ROS and directly producing lipid peroxidation and oxidative damage to DNA [47,72]; (b) indirect action, where ROS activate signaling pathways (e.g., MAPK pathway) and induce changes in cytokines and transcription factors such as IL-6, STAT3 and HIF-α1 [58,75]; and (c) generation of ROS by lung epithelial cells and alveolar macrophages after the induction produced by the VOCs present in the PM [72]. In general, the proposed mechanisms generate a state of cellular stress that, when maintained over time, results in the deregulation of signaling pathways corresponding to the expression of genes required for the response to pathological changes and the rectifying of the alterations in the respiratory system [9,72].

Another study in an animal model, where 3,4-benzopyrene was used as an exposure agent, showed bronchial inflammation and infiltration of many neutrophils and lymphocytes in the first week. After four weeks of treatment, deregulation of some genes (*p53, BCL2*), and local hyperplasia followed by metaplasia in bronchial mucosa and submucosa were observed [76]. The initial acute inflammatory response can protect the respiratory tract from irritating substances. On the other hand, a state of chronic inflammation can lead to a long-term decrease in respiratory function, as a result of tissue transformation (e.g., squamous metaplasia) producing potentially medium- and long-term malignant disease [77,78].

A strong correlation between pollution levels and the risk of lung cancer has been reported [79]. De Groot et al. [79] pointed out that an increase of 10 µg/m^3^ in the average concentration of PM_2.5_ with long-term exposure is associated with an 8% risk of death from lung cancer. In this long-term exposure experiment, two key factors in cellular toxicity resulting from exposure to PAHs can be considered [9,72]. The first factor is the formation of reactive metabolites that have demonstrated carcinogenic and mutagenic effects [72,80]. A second factor is the effective biological dose, which depends on the metabolization of PAHs by the complex (CYP-450), and is closely related to cellular toxicity [47,80]. Within the carcinogenic mechanisms, reactive epoxides (e.g., BPDE) covalently bind to DNA, resulting in the formation of adducts (PAH-DNA) [81,82]. These can generate nucleotide methylation, inactivation of enzymes, or the formation of bulkier adducts that interfere with DNA replication and repair processes [83], events that frequently occur at sites critical for the regulation of cell division [47,80]. The most affected cells are those with a high replication rate such as skin and lung tissue cells. For their part, it has been reported that lung cancer cells show multiple chromosomal breaks that imply loss of heterozygosity (LOH) in key genes such as *p53* and *RB* [83,84]. It is known that B[a]P epoxide adducts can be derived from LOH of the *p53* gene in bronchial epithelial cells, a mutation associated with lung cancer, and greater expression of the AhR and of xenobiotic metabolism enzymes that predispose the formation of reactive metabolites [85,86]. Additionally, there is a deregulation of signaling pathways that promote growth (e.g., PI3K/Akt, K-Ras and PTEN), and of pathways that inhibit it (e.g., P53, Rb, P14, STK11), and also in pathways related to cell apoptosis (e.g., Bcl-2, Bax/Fas) in lung tissue cells [83,87]. The metabolization of PAHs present in the PM is closely coordinated by genes of the CYP-450 complex and the AhR [88]. An interesting study in an animal model exposed to B[a]P revealed differential expression of more than 558 genes (e.g., *CYP1b1*,* CYP1A*,* AhR*), as well as changes in signaling pathways of xenobiotic metabolism, AhR activity, oxidative stress and p53 signaling [86].

In the context of epigenetic regulation, miRNAs have been widely studied for their regulatory role in the expression of multiple genes [24]. In 2004, it was shown that more than half of the miRNA genes are located in genomic regions associated with lung cancer [89] and it has been described that their deregulation could be involved in the carcinogenesis of lung tissue [89,90,91]. Various in vitro and in vivo studies have shown a significant number of miRNAs with deregulated expression after exposure to VOCs [28]. Additionally, it has been reported [28] that some metabolites (e.g., BPDE) derived from the metabolization of PAHs affect the maturation of miRNAs and do not allow their processing by biogenesis enzymes.

## 4. Biogenesis, Mechanisms of Actions and Regulation of miRNAs

**Figure 1:**
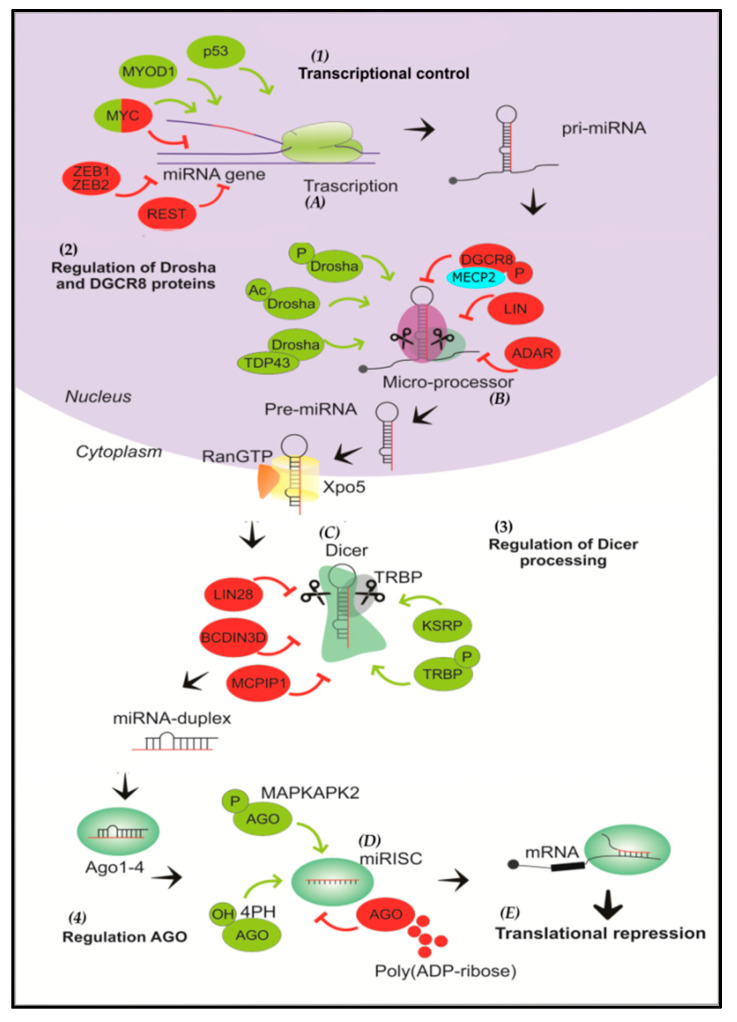
Overview of microRNA biogenesis and regulation. (1) miRNAs are initially transcribed by RNA polymerase II as pri-miRNAs that adopt a hairpin structure. (A) In the transcriptional control of the miRNA gene, the process can be positively regulated by p53, MYOD1, and MYC, and negatively regulated by ZEB1,2, REST and MYC. The transcription factor MYC can stimulate the expression of some miRNAs (e.g., the miR-17-92 oncogenic cluster), as well as inhibit the expression of several tumor suppressor miRNAs (e.g., miR-15). (2) Then, the Drosha/DGCR8 complex cleaves the pri-miRNA into a miRNA precursor and is transported to the cytoplasm by Exportin 5. (B) Drosha and DGCR8 are positively regulated by phosphorylation and acetylation reactions of Drosha, and through the interaction of Drosha and TDP43; on the other hand, phosphorylation of DGCR8 and the action of LIN and ADAR proteins negatively regulate the microprocessor. (3) The pre-miRNA is then cleaved by Dicer to form a miRNA duplex. (C) KSRP protein and TRBP phosphorylation positively participate in Dicer regulation, while the action of LIN28, BCDIN3D and MCPIP1 negatively regulate processing by Dicer. (D) One strand is then selected to function as a mature miRNA and is loaded into AGO to form the RNA-induced silencing complex (RISC). (4) Phosphorylation of AGO by MAPKAPK2 and hydroxylation of AGO2 by 4PH are positive regulators, whereas poly-(ADP-ribosylation) of AGO inhibits its activity. (E) Finally, the mature miRNA binds to the 3′UTR region of the target mRNA, resulting in translational repression and degradation.

miRNAs are a family of non-coding RNAs that participate in the regulation of gene expression [23]. Even though they represent only 3% of the human genome, it has been described that more than 60% of the coding genes in humans are regulated by miRNAs [92,93]. They participate in key biological processes such as inflammation, cell cycle regulation, and stress response, among others [23,94]. It has also been shown that deregulation in their expression is associated with a large number of human diseases, including cancer [27,95]. The transcription of miRNAs is carried out by RNA polymerase II (Figure 1), which results in a primary transcript (pri-miRNA) of ≈80 nucleotides which adopt a hairpin-shaped structure with a 5′ methylated portion and a 3′ polyadenylated tail [96,97]. miRNA genes can be located in intergenic regions, which can be individually transcribed from their promoters into a primary transcript (pri-miRNA) by RNA polymerase II [96], or they can be located in intronic regions and can be co-transcribed as part of the mRNA when their sequence is in the same orientation as the host gene [98,99]. In addition, it has been observed that some miRNAs have multiple transcription start sites [100]. After transcription, the (pri-miRNA) is processed by different enzymatic complexes. The enzyme Drosha/RNase III and its cofactor (DGCR8) process the (pri-miRNA) into a precursor of ≈65 nucleotides called pre-microRNA [97,101]. The pre-microRNA is exported from the nucleus to the cytoplasm, where its maturation is completed. Export is carried out by (EXP 5), responsible for transporting miRNAs from the nucleus to the cytoplasm through the nuclear pore in the presence of the Ran-GTP cofactor; when GTP is hydrolyzed, the complex is separated and the pre-miRNA is released into the cytosol [101]. The (pre-miRNA) is processed to an asymmetric duplex (miRNA/miRNA*) by the interface between Dicer and its cofactor TRBP, which contributes to the selection of the leading strand [102,103] and then the release of a small RNA duplex [101]. The RNA duplex, together with Argonaute (AGO), forms an effector complex, called the RNA-induced silencing complex (RISC) [104]. After the formation of the complex, the endonuclease (C3PO) cleaves the passenger strand (miRNA*), preserving the leader strand (mature RISC complex) [101,105]. Many alternative pathways of miRNA biogenesis have been described, where AGO binds directly to the small RNA duplex and activates it independently of the activity of Dicer or Drosha [104]. However, the vast majority of functional miRNAs follow the canonical pathway, since only around 1% of miRNAs mature through alternative pathways [101].

### 4.1. Mechanisms of Actions

MiRNAs induce silencing of gene expression by binding to complementary sequences of the target mRNA located in the 3′UTR region, a sequence named miRNA Recognition Element (MRE). This complementary region in the miRNA is called the seed region, and has a length of 2 to 8 base pairs, which allows for one miRNA to bind to several, even hundreds, of target mRNAs or a single mRNA [106,107,108]. If there is high complementarity between the miRNA and the mRNA, the latter is cleaved by ribonucleoproteins of the RISC complex. However, if the binding is partially complementary, deadenylation is triggered to induce rapid degradation of the mRNA [109,110]. Furthermore, several studies have indicated that some miRNAs can bind to the 5′ UTR region of mRNAs [111,112].

Although the general location of the union site is very important, there are also other factors that contribute to translational repression. These factors include the union site sequence, the number of complementary sites (within the mRNA) and the RNA local structure [113,114,115].

Also, it has been reported that translation inhibition occurs when this has started, as, for example, with lin-4 miRNA [116]. The MiRNA-RISC complex can bind to translationally active mRNA, reducing ribosomal initiation and producing destabilization of the native peptide, a mechanism that is mediated through components of the RISC complex (Ago 1, 3 and 4 proteins) [116,117].

In cancer, miRNAs function as regulatory molecules, acting as oncogenes or tumor suppressors. Moreover, miRNAs can have a dual function, one miRNA being able to behave as an oncogene or a tumor suppressor, because its targets are tissue specific. For example, miR-221 and miR-222 can behave as oncogenes by inhibiting the expression of p27 in various types of tumors (thyroid, glioblastoma, prostate, lung, etc.) [118,119,120] and also as tumor suppressors by inhibiting proto-oncogen *KIT* in erythroleukemia [121]; miR-20a is a tumor suppressor in hepatocellular carcinoma [122] oral squamous cell carcinoma [123] and suppressor cells of myeloid origin (MDSCs) [124], and a potential oncogene in gliomas [125], colon cancer [126] and gastric carcinoma [127].

Additionally, cytoplasmic processing bodies, or P-bodies, contain a high concentration of enzymes required for mRNA turnover and translational repression. Some studies provide evidence that the mRNAs silenced by miRNAs are localized to P-bodies for storage or degradation [128,129,130]. On the other hand, in 2010, the first evidence was provided of a direct miR–protein binding by showing in chronic myelogenous leukemia that miR-328 can sterically bind to hnRNP E2, an inhibitor of the transcription factor CEBP-a [131]. This direct interaction between a miR and a protein, through miReceptors, also opens a new field of research where miRNAs can be transported intercellularly within vesicles mediating paracrine and endocrine interactions. The discovery that miRs can function as ‘hormones’, and can bind to and activate a receptor has changed our understanding of how miRs operate [132].

### 4.2. Regulation of miRNAs

#### 4.2.1. Regulation at the Transcriptional Level

Some transcription factors have been shown to exert positive or negative regulatory action over the biogenesis of miRNAs (p53, MYC, ZEB, MYOD1) [133,134]. Changes in the methylation of tumor suppressor miRNAs (e.g., miR-127 and miR-124a) have been reported [94,135].

#### 4.2.2. Regulation at the Post-Transcriptional Level

The processing of pri-miRNA by the microprocessor complex has been identified as a positive regulation event. This can include autoregulation between Drosha and its cofactor DGCR8 (a condition that provides better homeostatic maintenance of the microprocessor), phosphorylation and acetylation, which provide nuclear localization and stability to Drosha, respectively, and deacetylation and phosphorylation, which improve affinity/stability of pri-miRNA with DGCR8 [101,136]. Additionally, it has been described that the terminal loops of the pri-miRNA are enriched with Cis elements that recruit regulatory proteins (R-SMADs and p53) to promote the activity of the microprocessor [101]. On the other hand, there are negative regulatory events, including the phosphorylation of MECP2 which sequesters the Drosha cofactor DGCR8, inhibiting its enzymatic activity [137]. The proteins LIN28A and LIN28B can bind to the terminal loop of the pri-miRNA and suppress the processing mediated by Drosha and Dicer, which are also classified as regulatory events [101,138].

#### 4.2.3. Regulation at the Dicer Level

The KSRP protein that facilitates the processing of several pre-miRNAs by Dicer through the interaction with the terminal loop, as well as the phosphorylation of the protein (TRBP) linked to signaling, have been identified as positive regulatory events. MAPK/ERK leads to a state of regulation which promotes miRNA growth. As negative regulatory events, the LIN28 protein can specifically bind to the terminal loop of several pre-miRNAs to interfere with processing by Dicer [101]. Uridylation of the structure of pre-miRNA can also interfere with processing by Dicer [101]. The enzymes adenosine deaminase acting on RNA (ADARs) have been also described to modify certain pre-miRNAs by reducing the processing mediated by Dicer [101,103]. In addition, the methylation at the 5′-monophosphate end of the pre-miRNA exerted by methyl transferase BCDIN3D can also interfere with Dicer-mediated processing [139]. Interestingly, BCDIN3D seems to phosphodimethylate pre-miR-145, which induces a decreased processing of pre-miRNAs by Dicer in vitro [139].

#### 4.2.4. Regulation at the Level of AGO Proteins

AGO proteins can be modulated by numerous modifications. As a positive regulatory event under the condition of tissue hypoxia, the prolyl 4-hydroxylation of human AGO2 by type I prolyl-4-hydroxylase (4PH) increases its stability [140]. Phosphorylation at residue Ser 387 of AGO2 through signaling (MAPKAPK2) results in the localization of bodies for mRNA degradation [101]. As a negative regulatory event in the face of a state of cellular stress, human AGO proteins are subject to poly ADP-ribosylation, a process that inhibits their activity [101]. A study showed that the knockout of Drosha and Dicer caused a marked decrease in the levels of miRNAs; however, in XPO5-knockout cells the levels of miRNAs were not significantly altered, leading to the conclusion that the activity of Drosha and Dicer is critical for biogenesis and that the function of (XPO5) can be complemented by alternative mechanisms [141].

### 4.3. Other Regulatory Mechanisms

#### 4.3.1. Regulation Based on Genomic Localization

Multiple miRNA genes are found in fragile sites in the genome [142], including structural and functional genomic alterations such as mutations, insertions, deletions, and changes in the number of copies of particular regions (amplifications) [142]. Furthermore, it has been seen that fragile sites in the genome are very dense in miRNA genes [143].

#### 4.3.2. Regulatory Biogenesis Proteins

Certain proteins as RNA-binding proteins (RBPs) are key in the regulation of the biogenesis and functional activity of miRNAs, depending on the metabolic conditions [144].

#### 4.3.3. Alternative Splicing

A competitive interaction has been identified for the transcription of an exonic region located between exon–intron junctions by the spliceosome complex and the miRNA microprocessor complex [145]. When the spliceosome machinery does not recognize the internal exon, the microprocessor components bind to the transcription region of the pri-miRNA. On the other hand, when the spliceosome machinery recognizes the internal exon, it is processed into a protein transcript, a regulatory mechanism that specifically controls the maturation of some miRNAs [145].

## 5. MicroRNAs as Biomarkers of Acute Inflammatory Response to PAH Exposure in the Respiratory Tract

Multiple studies have shown a significant number of miRNAs are deregulated post-exposure to environmental pollutants, suggesting that miRNAs are sensitive indicators of the effects of contamination [28,29]. In vitro and in vivo studies have shown that a large number of miRNAs are deregulated post exposure to VOCs, whose targets are mainly related to oxidative stress and the inflammatory response [86,146,147].

Jardim et al. [148] analyzed the expression profiles in bronchial epithelial cells obtained by bronchoalveolar lavage from healthy people exposed to DEP. The results showed six upregulated miRNAs (miR-513c, miR-513b, miR-513a-5p, miR-923, miR-494, and miR-338-5p) and six downregulated miRNAs (miR-26b, miR-27a, miR-31, miR-96, miR-135b, and miR-374a). Additionally, in the in silico analysis, a relationship was found with important signaling pathways (IL-8, NF-kB, TGF-β y CXCR-4) [148]. Likewise, Wu et al. studied the synergistic effects of PAHs on lung fibrosis mediated by the miR-30c-1-3p/transforming growth factor β II receptor (TGFβR2) axis. This study suggested that TGFβR2 was a target of miR-30c-1-3p and that miR-30c-1-3p could act as a negative regulator in B[a]P-induced pulmonary fibrosis by targeting TGFβR2 [149].

On the other hand, the study of Bleck et al. [150] found that DEP and environmental PM increase the expression of thymic stromal lymphopoietin (TSLP), a proinflammatory cytokine linked to adaptive immunity disorders [151] in human bronchial epithelial cells (pHBEC) through a mechanism that includes hsa-miR-375, with a consequent regulatory effect on AhR mRNA [150].

PAHs and their derivatives, such as B[a]P/B[a]PDE, are potent carcinogens that actively promote inflammation and lung cancer [152]. In the study by Huang et al. [153], both in vitro and in vivo, the environmental compound B[a]P/B[a]PDE decreased the expression of the PHLPP2 (PH Domain and Leucine Rich Repeat Protein Phosphatase 2) protein, an important tumor suppressor that regulates the Akt signaling pathway [154], which is related to the positive regulation of proinflammatory markers such as NFAT/NFκB, TNFα and COX-2. As an essential mechanism in lung carcinogenesis, miR-205 was additionally found to inhibit the translation of the PHLPP2 protein by binding to the 3′-UTR region of PHLPP2 after exposure to B[a]PDE [153].

Rider et al. [155] showed that expression of miRNAs and specific genes associated with the immune response was deregulated in bronchial cells exposed to DEP or allergens. An increase in the expression of the miRNAs miR-21-5p, miR-29a-3p, miR-29b-3p, miR-30d-5p, miR-223-3p and miR-4454, and a decrease in the expression of miR-34c-3p, miR-98, miR-125b-5p, miR-140-5p, miR-181a-5p, miR-181b/d-5p, miR-197-3p, miR-331-3p, miR-423-3p, and miR-425-5p was caused by DEP in human bronchial epithelial cells obtained by bronchoalveolar lavage. An in silico analysis was able to correlate potential highly regulated target genes (*CD24*,* GZMA*,* HFE*,* IKZF3*,* PML*, and *SLAMF7*) and four poorly regulated genes (*CASP2*,* CX3CL1*,* IRF8*, and *TLR5*) [155].

Subsequently, a study by Halappanavar et al. [86] analyzed the effect of oral exposure to B[a]P in mice for three consecutive days. The study demonstrated adduct formation in the DNA of lung tissue, highlighting the presence of 269 genes with deregulated expression (*p* < 0.05), especially CYP1A1, CYP1B1, Fmo3 and AhR. On the other hand, seven highly expressed miRNAs were observed (miR-34c, miR-34b-5p, miR-29b, miR-141, miR-199a-5p, miR-125a-5p, and miR-200c), and six miRNAs were downregulated (miR-122, miR-142-3p, miR-144, miR-142-5p, miR-150, and miR-451). From the bioinformatics analysis, some (miR-150, miR-34c, miR-29b, miR-34b-5p, miR-142-5p, and miR-122) were predicted to interact with genes involved in the cell cycle, apoptosis, tumor suppressor activity, and inflammatory response [86]. Likewise, a study led by Chen et al. [156] reported that α4, the regulator of protein phosphatase 2A (PP2A) which is involved in tumorigenesis, was highly expressed in human bronchial epithelial cells that had been chemically transformed with B[a]P (HBERT-B[a]P). Functional studies determined that miR-34b suppressed the expression of α4, negatively regulating its expression, thus playing a role in tumor suppression [156].

It has recently been described that carbon nanoparticles (CBNPs) transport PAHs, in addition to inducing the formation of ROS, IL-6 and fibronectin in mouse lung tissue [157,158]. Two studies evaluated the effect of CBNPs and black carbon nanoparticles (nCB), with residential smoke (particulate matter emitted from the combustion of different biomass fuels) and diesel vehicle emissions being the main generators of the nanoparticles, respectively [40,159]. Bourdon et al., [146] analyzed the effect of exposure to nCB in mouse lung tissue, showing that three miRNAs were highly expressed (miR-21, miR-146b and miR-135b) (*p* ≤ 0.05). Target analysis revealed no concomitant changes in established and predicted targets of miR-135b (Adamts9, Bmper, Klf4, Cxcl12 and Rrbp1 targets), miR-21 (PTEN, Pdcd4, Tpml, and Cxcl10 targets), or miR-146b (Klf4 and Uhrl targets) [146]. Similarly, Lu et al. [160] analyzed the effect of nCB in mice, observing an increase in alveolar space and total lung volume with an accumulation of macrophages and neutrophils in the airway, finding that miR-22 has potential pro-inflammatory action because miR-22 *knockout* mice showed less inflammation after nCB exposure compared to wild type mice (*p* < 0.05), which could be related to its regulatory action on histone (HDAC4) and IL-6 [160].

In mouse lung tissue, Wang F. et al. [161] analyzed the effect of exposure in vivo to VOCs in bronchoalveolar lavages. They found a significant increase in IL-8, LDH, and NOS and a decrease in glutathione (GSH) (*p* < 0.05), identifying multiple deregulated miRNAs, of which six were overexpressed (miR-1187, miR-125a-3p, miR-125b-5p, miR-466c-5p, miR-5105 and miR-3472), related to signaling pathways associated with adhesion molecules, chemokines and growth factors (jak-STAT) [161]. In a different study, Wang C.C et al. found a significant increase in lymph nodes in the lung tissue, associated in part with the increase in the levels of proinflammatory cytokines MIP-2, TNF-α, IL-6 and IL-1β induced by B[a]P in mice, which was associated with repression of miR-101 and a consequent increase in IL-1β and Lin28B [162]. This evidence directly links the deregulation of miRNAs with PAHs (Table 2) in important signaling pathways involved in inflammation, cellular stress, proliferation and apoptosis.

## 6. Differential Expression of miRNAs in Lung Cancer in Response to PAH Exposure

Multiple studies have analyzed the differential expression of miRNAs in neoplastic tissue induced by exposure to carcinogens [163,164]. Shen et al. performed one of the first studies on the expression of miRNAs in cell lines derived from lung cancer post exposure to anti-BPDE [165]. Fifty-four downregulated miRNAs were identified in transformed bronchial epithelial cells (16HBE-T) compared to controls. Three members of the miR-17-92 clusters (miR-17-5p, miR-20a, and miR-92) showed significantly high abundance in 16BHE-T cells versus miR-21, miR-141, miR-27a, miR- 27b, miR-16, and miRNAs from the let-7 family. The putative target for miR-10a, HOXA1 mRNA (homeobox protein A1) was upregulated 3-9-fold in 16HBE-T cells compared to controls [165]; HOXA1 is a protein that is highly expressed in non-small-cell lung cancer (NSCLC) and is associated with lower survival in patients [166].

Another study performed by Liu et al. [167] showed that miR-494 expression increased while PTEN protein seemed to be decreased in malignant transformed 16HBE-T cells. Moreover, the decreased expression of miR-494 increased caspase-3/7 activities in transformed 16HBE cells [167]. In addition, miR-22 was shown to be highly expressed in transformed cells, concomitant with the downregulation of the tumor suppressor gene PTEN, which suggests that miR-22 induces PTEN repression through a translational mechanism in NSCLC [168,169].

Duan et al. [170] analyzed the expression profiles of miRNAs in transformed cells of mouse bronchial epithelium after exposure to B[a]P, finding a high expression of miR-320 and miR-494 and suggesting a possible mechanism of regulation of the cell cycle through CDK6 [170], a protein involved in the response to DNA damage by carcinogenic chemicals [171]. In addition, Barkley et al. found that the upregulation of Cdc7 kinase correlates with the downregulation of miR-29a as a response to DNA damage [172]. The enforced miR-29a expression could reduce the accumulation of Cdc7 kinase induced by the environmental genotoxin, benzo[a]pyrene dihydrodiolepoxide (BPDE) [172].

The study by Zhao et al. [173] carried out in 16HBE-T cells using anti-BPDE showed that miR-506 was significantly repressed, with an inverse correlation with the proto-oncogene N-Ras expression, whereas on restoring the expression of miR-506, a decrease in cell proliferation was observed [173]. The same group confirmed a lower expression of miRNA-542-3p in the same in vitro model, which led to a significant decrease in the proliferation capacity and malignant features in the 16HBE-T cells. Both studies demonstrate the potential role of miR-506 and miR-542-3p as tumor suppressor genes contributing to malignant transformation after the exposure to anti-BPDE when they are repressed [174].

On the other hand, Li et al., [175] studied the expression profiles of miRNAs in human bronchial epithelial cells (HBER) treated with B[a]P, identifying twelve deregulated miRNAs. Here, it was observed that the miR-638 overexpression worsened the cellular DNA damage induced by BaP, which could be related to the upregulation of the tumor suppressor*BRCA1* (breast cancer gene 1), and that aberrant expression of miR-638 confers the ability of cells to adapt to environmental stress or maintain the state of malignancy [175]. Additionally, Pan et al. [176] found a significantly decreased expression of miR-144 in the lung cancer tissues of patients from Yunnan province (China), a region characterized by high pollution from coal combustion [177], demonstrating through in vitro and in vivo studies that miR-144 has a potential tumor-suppressor role by regulating somehow the expression of *ZEB1-ZEB2* [176]. This is very similar to the functions described for miR-622, which has a potential role as a tumor suppressor through the regulation of *KRAS*, and which is repressed in the 16HBE-T cells transformed with anti-BDPE [178]. Moreover, Jiang et al. [179] found forty-three upregulated miRNAs and thirty-three repressed miRNAs in BEAS-2B cells (human bronchial epithelial cell line) transformed with B[a]P. In this study, the miR-138-5p repression was associated with the PDGFRB/IncRNA-RGMBAS1/circ-ZNF292 pathway, which could be involved in malignant transformation [179].

Yadav et al. [180] demonstrated that the protein Ubiquilin (UBQLN) provides protection against tissue injury and oxidative stress produced by exposure to DEP. Their previous studies reported that the loss of UBQLN proteins is associated with the progression of lung cancer [180,181], confirming that the low expression of UBQLN1 and UBQLN2 caused by miR-155 (as demonstrated by luciferase assays) promotes greater invasion and migration in lung cancer tumors [180]. Table 3 summarizes the microRNAs deregulated by exposure to PAHs in cell lines of lung cancer or neo-plastic tissues or benzo(a)pyrene-transformed bronchial epithelial cells. In this regard, Li et al. indicated that Benzo[a]pyrene (BaP) is a potent carcinogen and miRNAs may play an important role in BaP-involving carcinogenesis. Repression of miR-152-3p, miR-142-5p and miR-211-5p was involved in the upregulation of the activated leukocyte cell adhesion molecule (ALCAM), which was upregulated in the BaP-transformed 16HBE cells (THBEc1), and may be a key molecule for THBEc1 cells to gain and maintain the malignant phenotype [182].

Recently, Tian et al. reported that B[a]PDE induces the expression of the ATG7 (Autophagy Related 7) protein, contributing to malignant transformation in bronchial epithelial cells through the degradation of the DNMT3B protein (DNA Methyltransferase 3 Beta) and methylation of the miR-494 promoter [183].

**Table 3:** MicroRNAs deregulated by exposure to PAHs in cell lines of lung cancer or neoplastic tissues or benzo(a)pyrene-transformed bronchial epithelial cells.

Pollutant	Tissue/Cell Type	microRNA Expression: Up/Down Regulation	Potential Target/Pathway	Reference
anti-BPDE	Cell line (16HBE-T)	Down: miR-10a	HOXA1	[165]
anti-BPDE	Cell line (16HBE-T)	Up: miR-494	PTEN	[167]
anti-BPDE	Cell line (16HBE-T)	Up: miR-22	PTEN	[168]
anti-BPDE	Cell line (16HBE-T)	Up: miR-622	KRAS	[178]
B[a]P	Mouse lung tissue	Up: miR-320, miR-494	CDK6	[170]
B[a]P	HBE-1A1 cells	Down: miR-3173-5p Up: miR-1343-3p, miR-27a-5p, miR-219a-1-3p	CDKN1A, MAPK13, ALDH1A3 SMAD2 CYP1A1 IL1A and IL1B	[184]
B[a]P	BEAS-2B cells	Up: miR-377-3p	Wnt/β-catenin pathway via EGR1 downregulation	[185]
Β[a]P	Primary lung tumors	Down: miR-34b	α4	[156]
B[a]P	Cell line (BEAS-2B)	Down: miR-138-5p	PDGFRB, IncRNA RGMB-AS1 and circ-ZNF292	[179]
B[a]P	Cell line (16HBE-T)	Down: miR-152-3p, miR-142-5p and miR-211-5p	ALCAM	[182]
B[a]P	Mouse lung tissue	Down: miR-101	IL-1β and Lin28B	[162]
BPDE	Cell line (A-549)	Down: miR-29a	Cdc7	[172]
anti-BPDE	Cell line (16HBE-T)	Down: miR-506	N-Ras	[173]
anti-BPDE	Cell line (16HBE-T)	Down: miR-542-3p	Cellular cycle	[174]
B[a]P	Cell line (HBER)	Up: miR-638	BRCA1	[175]
nCB	Cell line (A549)	Down: miR-144	ZEB1	[176]
BPDE	Cell line (BEAS-2B), (A549)	Up: miR-205	PHLPP2	[153]
DEP	Cell line (A549), (H358)	Up: miR-155	UBQLN1, UBQLN2	[180]
NaP, Acy, Ace, Ant, Flu, Pyr, BaA, Chr, BbF, BkF, B[a]P and DBA	Cell line A549	Up: miR-222, miR-210, miR-101, miR-34, miR-93 and miR-200a	NFKB, MYCN, CDKN1A, CDKN1B, RAD52, BAG1	[38]

anti-BPDE: anti-benzo[a]pyreno-7, 8-diol-9,10-epoxide; A549: human lung carcinoma cell line; B[a]P: Benzo[a]pyrene; BPDE: Benzo[a]pyrene-diolepoxide; NaP: Napthalene; Acy: Acenapthylene; Ace: Acenapthene; Ant: Anthracene; Fl: Fluorene; Pyr: Pyrene; BaA: Benzo(a)anthracene; Chr: Chrysene; BbF: Benzo(b)fluoranthene; BkF: Benzo(k)fluoranthene; DBA: Dibenzo(a,h)anthracene; DEP: Diesel engine emission particles; nCB: Black carbon nanoparticles; H358: human bronchioalveolar carcinoma cell line; 16HBE-T: Transformed human bronchial epithelium cell line; HBER: human bronchial epithelial cells expressing the oncogenic H-Ras allele.

## 7. Discussion

Prolonged exposure to environmental pollution is a risk condition for the development of lung disease [50]. Among the pollutants present in PM, PAHs generate and promote an inflammatory state [31,64] and their reactive metabolites (e.g., BPDE) produce adducts in DNA that interfere with DNA replication and repair processes, events that frequently occur in critical sites for the regulation of the inflammatory response and regulation of the cell cycle [83,85]. Among the short-term effects, studies in the school population have reported deterioration in lung function and an increase in the cases of airway inflammation [57,186], and a greater risk of developing lung cancer in people with sustained exposure over time [11,48].

In recent years, it has been shown that the expression of miRNAs can be deregulated in respiratory diseases associated with environmental pollution [28,29], being potentially useful as biomarkers of exposure. MiRNAs are particularly stable, and laboratory detection methods are sensitive enough for the study of expression profiles [187,188]. Multiple overexpressed miRNAs have been described following acute exposure to PAHs, such as miR-513c, miR-513b, miR-513a-5p, miR-923, miR-494 [148], miR-375 [150], miR-205 [153], miR-21-5p, miR-29a-3p, miR-223-3p [155], miR-21, miR-135b, miR-146b [146], miR-22 [160], miR-1187, miR-125a-3p, miR-125b-5p, miR-466c-5p, miR-5105, and miR- 3472 [161]. In contrast, other miRNAs are downregulated, such as miR-26b, miR-27a, miR-31, miR-96, miR-135b, miR-374a [148], miR-122, miR-144, miR-150, miR-45 [86], miR-98, miR-125b-5p, miR-140-5p, miR-331-3p, miR-423-3p, miR-425-5p [155], miR-222, miR-210, miR-101, miR-34, miR-93, and miR-200a [38].

Two of the miRNAs that are most frequently deregulated in inflammation [189,190] post exposure to DEP and CBNPs are miR-223-3p [155] and miR-146 [146]. In most studies reviewed, direct and indirect association (in vitro and in vivo) with pro-inflammatory targets was observed [86,148,155] with cytosine and chemokine signaling pathways [150,153,160,161]. On the other hand, the overexpression of miR-494 [148,167] and miR-205 [153]) confers a role on them as biomarkers of exposure to DEP and BPDE. Recently, it has been shown that miRNA-205 can promote an inflammatory response through the regulation of Domain containing copper metabolism (COMMD1) in cancer cells [191]. The findings of Thum et al. [192] and Yanaihara et al. [193] also highlight the role of miR-205 after cigarette smoke exposure. Interestingly, miR-125b—which is deregulated after exposure to DEP [155], VOCs [161] and PAHs [29]—is associated with the regulation of macrophages, where its expression is decreased in alveolar macrophages after exposure to PAHs [194,195].

Studies of miRNAs circulating in blood have shown that miR-21 is highly expressed in lung tissue upon exposure to DEP and CBNPs [146,155], and is especially present in peripheral blood in workers at an electric furnace steel plant [196]. This indicates that miR-21 plays a key role in mediating inflammation and the negative regulation of the pro-inflammatory response [197], and is one of the most studied miRNAs due to the large number of biological processes and cell lines in which it is involved.

Interestingly Ulivi et al. explored the prognostic value of circulating miRNAs (c-miRNAs) in patients with resected early-stage non-small-cell lung cancer; they found that miR-21 and mir-205, in addition to miR-26a-5p, miR-126-3p and miR-130b-3p, were significantly associated with disease-free survival in squamous cell carcinoma, and four (miR-130b-3p, miR-26a-5p, miR-126-3p, and miR-205-5p) remained significantly associated with overall survival [198]. Similarly, Hu et al. studied circulating microRNAs in patients with lung adenocarcinoma and squamous cell carcinoma in the early stage of the disease, where eleven serum miRNAs (miR-486, miR-22, miR-30d, miR-21, miR-26b, let-7i, miR-378, miR-1, miR206, miR-146b, miR-499, let-7a and lte-7g) were found to be altered more than five-fold between longer-survival and shorter-survival groups [199].

This evidence indicates that miRNAs play a central role in the regulation of signaling pathways involved in inflammation [194], a condition that is linked to exposure and that promotes lung cancer progression [75,200], as well as in early lesions in patients with lung cancer [199]. Studies in cell lines derived from neoplastic lung tissues and treated in vitro with B[a]P, anti-BPDE and DEP, showed that those highly-expressed miRNAs are miR-494 [165], miR-22 [168], miR-320 [170], miR-638 [175], miR-207, miR-290, miR-291a-3p, miR-291a-5p, miR-376b, miR-434-3p, miR-219 [201], miR-205 [153] and miR-155 [180], whereas miR-10a [165], miR-506 [173] miR-542-3p [174], miR-29a [172], miR-433-5p, miR-292-5p, miR-291b-p [201] and miR-144 [176] are downregulated. Most of the deregulated miRNAs are closely linked to regulatory pathways associated with the expression of tumor suppressor genes [167,168] and oncogenes [173], which participate in cell cycle control mechanisms [172] and tissue repair [175,180]. In fact, the oncogenic miRNA-17-92 cluster and tumor suppressors of the let-7 family [165] are well known for their contribution to lung cancer development [202]. Furthermore, miR-22, miR-494 and miR155 are highly expressed after exposure to anti-BPDE and DEP [167,168,180], which correlates with studies where they are attributed as markers of progression in lung cancer [166,193,203]. Similarly, miR-22 has been implicated in breast cancer progression [204], epithelial ovarian cancer [205], and in prostate cancer, due to its interaction with PTEN [206], suggesting its role as a tumor suppressor [207]. On the other hand, miR-494 is postulated as a therapeutic target in the progression of lung cancer, behaving as an oncogene due to its interaction with PTEN, and, although information is more scarce, miR-155 has been reported in blood as a potential diagnostic and prognostic biomarker in lung cancer [193,208]. Interestingly, four miRNAs that are deregulated in neoplastic cell lines were also deregulated in tissue, specifically miR-21 [160,168], miR29a [155,172], miR-144 [86,176], and miR-205 [153]. On the other hand, the expression of miR-638 was correlated with exposure to 1-hydroxypyrene (1-OH), a hydroxylated compound derived from the metabolization of PAHs [209]; in vitro, its expression rises rapidly after two hours of exposure to B[a]P, and it can be re-established after 24 h of ending the exposure. Likewise, in chronic benzene poisoning (CBP) patients compared with non-exposed controls, the plasma level of miR-638 was significantly upregulated [210].

Some of the difficulties and differences in the results of the various studies may be associated with the study design, exposure dose, model (in vitro and in vivo), experimental miRNA analysis techniques, and the quality of the samples that allow a reliable estimation of miRNA expression profiles [188,211]. Furthermore, exposure to different compounds may in some cases not be representative of local air pollution; only a few studies have explicitly emphasized this point [150,153,161] and they indicate having used a dose equivalent to the air quality of the local city or the representative dose of the emission source. Furthermore, in vitro exposure may not always be representative of what would normally occur in people or in in vivo studies, since in vitro cells are isolated without their normal cellular microenvironment [77,212]. Moreover, anatomical factors and interactions between biological systems attenuate the impact of contaminants [213,214]. Another relevant aspect is the exposure time; in most studies, deregulation of miRNAs post exposure is reflected after 24–48 h, and in some cases such as miR-638 [175], miR-320 and miR-494 [170] it is observed that their basal expression level is restored after 24 h post exposure, showing a state of transient deregulation in response to acute exposure. On the other hand, in the vast majority of epidemiological studies related to exposure to PAHs, estimates of health risk may be uncertain, because environmental contaminants are usually complex mixtures of PAHs added to other types of contaminants that can also generate a detrimental effect [11,215], such as volatile metals [196], pesticides [216] and other nanoparticles [217,218]. Thus, the expression of miRNAs may be different from the reactive metabolite and the primary contaminant, as occurs with miR-205, which is deregulated in the presence of the reactive metabolite BPDE but not against the primary contaminant B[a]P [153]. Therefore, to investigate exposure to PAHs, it is important to characterize the composition of the local air where individuals live and work [219,220], as well as to consider the racial disparity of the populations studied. Differences between ethnic groups are determined by genetic, environmental and cultural factors, factors that can be independent of each other, or that can interact in a dynamic and synergistic way [221].

## 8. Concluding Remarks

MiRNAs have emerged as an important family of molecules with promising perspectives as biomarkers, since they harbor essential characteristics such as stability due to their resistance to the activity of Ribonucleases (RNases)reproducibility, and high tissue specificity among individuals [222]. Thus, it is very important that their validation as biomarkers is explored in a greater number of studies and clinical trials that allow their sensitivity and specificity to be determined, using different experimental contexts and genetic and epigenetic profiles, with the aim of establishing risk groups in a timely manner to prevent disease, guide therapeutic decisions, and predict disease evolution.

Added to this, with the rapid development of gene-chip and high-throughput sequencing technologies, access to multiple biological and microRNA data and artificial intelligence and algorithm development, together with the development of microRNA-based machine learning models could be used to enhance the diagnostic accuracy of a plethora of diseases, while at the same time substituting or minimizing the use of invasive diagnostic means [223]. New calculation methods have recently been developed to predict associations between miRNA and diseases [224]. Thus, artificial intelligence has demonstrated superior efficacy in generating disease phenomapping, early warning systems, risk prediction, automated processing and the interpretation of imaging, and increasing operational efficiency [225]. It has recently been published that an algorithm managed to identify people at higher risk of suffering from pancreatic cancer up to three years before diagnosis; this approach only used clinical data from the patients [226].

Many of the studies presented here are derived from lung tissue and samples obtained by bronchoalveolar lavages, which require invasive procedures. In this context, the search for easily accessible, stable and non-invasive markers such as miRNAs is currently of great interest, and this is why circulating miRNAs have been the focus of key studies, where several of them have been shown to be deregulated in peripheral blood post exposure to PAHs [227], PM [196,228] and VOCs [229]. Furthermore, they have been linked to oxidative stress and the inflammatory response and, interestingly, their expression in vitro has been correlated with that of peripheral blood samples (miR-21 [146,196], miR-29a [172,228] and let-7 [165,228]).

Such evidence thus highlights the fact that miRNAs are promising biomarkers in evaluating the degree of exposure of individuals to PAHs, as well as their association with inflammation and lung cancer [230].

## Figures and Tables

**Table 2:** MicroRNAs deregulated in non-tumorigenic human cell lines and tissues, and normal mouse lung tissue on exposure to PAHs.

Pollutant	Tissue/Cell Type	Regulation Up/Down	Potential Target/Regulatory Mechanism	Reference
DEP/PM	Human bronchial cells	Up: miR-513c, miR-513b, miR-513a-5p, miR-923, miR-494, miR-338-5p Down: miR-26b, miR-27a, miR-31, miR-96, miR-135b, miR-374a	Diana not validated CD274, immunology Fragment of 28S RNA PTEN ABC Transporters P53, TGF-β Apoptosis Inflammation DICER, ATM	[148]
DEP/PM	Primary pFBEC cells	Up: miR-375	TSLP, AhR, immunity	[150]
BPDE	BEAS-2B cells and mouse	Up: miR-205	PHLPP2	[153]
DEP/PM	Human lung tissue	Up: miR-21-5p, miR-29a-3p, miR-29b-3p, miR-30d-5p, miR-223-3p, miR-4454 Down: miR-34c-3p, miR-98, miR-125b-5p, miR-140-5p, miR-181a-5p, miR-181b/d-5p, miR-197-3p, miR-331-3p, miR-423-3p, miR-425-5p	Diana not validated	[155]
B[a]P	Mouse lung tissue	Up: miR-34c, miR- 34b -5p, miR-29b, miR-141, miR-199a -5p, miR-125a-5p, miR-200c Down: miR-122, miR-142-3p, mi-144, miR-142-5p, miR-150, miR-451	Oxidative stress, Xenobiotic metabolism, inflammation	[86]
B[a]P	BEAS-2B cells	Up: miR-30c-1-3p	TGFβR2	[149]
CBNPs	Mouse lung tissue	Up: miR-21, miR-135b, miR-146b	Cellular apoptosis, oxidative stress, inflammation	[146]
nCB	Mouse lung issue cells	Up:miR-22	HDAC4	[160]
VOCs	Mouse lung tissue	Up: miR-1187, miR-125a-3p, miR-125b-5p, miR-466c-5p, miR-5105, miR- 3472	Cell cycle, apoptosis, inflammatory response	[161]

BPDE: Benzo[a]pyrene-diolepoxide; B[a]P: Benzo[a]pyrene; CBNPs: carbon nanoparticles; nCB: Black carbon nanoparticles; BEAS-2B: human bronchial epithelium cells; VOCs: volatile organic compounds; pHBEC: primary cells of human bronchial epithelium; HDAC4: Histone deacetylase 4; DEP/PM: Diesel engine emission particles in particulate matter.

## Data Availability

No new data were created or analyzed in this study. Data sharing is not applicable to this article.

## Abbreviations

3′UTR	3′ Untranslated Region	IL-8	Interleukin 8
A-549	Human lung carcinoma cell line	IL-1α	Interleukin 1 alpha
Ace	Acenapthene	IL-1β	Interleukin 1 beta
Acy	Acenapthylene	K-RAS	KRAS protein oncogene
ADAR	Adenosine deaminase-specific RNA	LDH	Lactate dehydrogenase
AGO	Argonaut protein	LIN28A	Lin-28 gene homolog A
AhR	Aryl hydrocarbon receptor	LIN28B	Lin-28 gene homolog B
AK	Adenylatekinase	LOH	Loss of heterozygosity
AKT3	Serine/threonine kinase 3	MAPK	Mitogen-activated kinase
ALCAM	CD166 Antigen	MAPKAPK2	Protein kinase-activated kinase 2
ALDH1A3	Aldehyde dehydrogenase 1 member A3	MECP2	MethylCpG binding protein
Ant	Anthracene	MIP-2	Macrophage inflammatory protein 2
Anti-BPDE	Anti-benzo[a]pyrene-7,8-diol-9,10-epoxide	MYCN	Proto-oncogene
AP-1	AP-1 transcription factor	MYOD1	Myoblast determination protein 1
ARF	ADP ribosylation factors	NaP	Napthalene
BaA	Benzo(a)anthracene	nCB	Black carbon nanoparticles
B[a]P	Benzo[a]pyrene	NF-KB	Nuclear factor kappa B
BAG1	BAG family molecular chaperone regulator 1	Pyr	Pyrene
BbF	Benzo(b)fluoranthene	PDGFRB	Platelet-derived growth factor beta receptor
BCDIN3D	BCDIN3 domain containing RNA methyltransferase	PP2A	Protein phosphatase 2A holoenzyme
BEAS-2B	Human respiratory epithelium cells	RAD52	RAD52 homolog
BkF	Benzo(k)fluoranthene	RNA	Ribonucleic Acid
BPDE	Benzo[a]pyrene-diolepoxide	ROS	Reactive oxygen species
BRCA1	Breast cancer gene 1	R-SMADs	SMAD protein activated receptor
CBP	Chronic benzene poisoning	SCLC	Small-cell lung cancer
COMMD1	Domain containing copper metabolism	TGFβR2	Transforming growth factor beta 2 receptor
CDKN1A	Cyclin-dependent kinase 1A inhibitor	THP-1	Cell line derived from acute monocytic leukemia
CDKN1B	Cyclin-dependent kinase 1B inhibitor	TNF- α	Tumor necrosis factor alpha
Chr	Chrysene	TRBP	RISC complex binding protein
CXCR-4	Chemokine receptor type 4	TSLP	Stromalthymic lymphopoietin
C3PO	C3PO ribonuclease	UTR	Untranslated Region
CBNPs	Carbon nanoparticles	XPO5	Exportin 5
COPD	Chronic obstructive pulmonary disease	NOS	Nitric oxide synthase
COX-2	Cyclooxygenase 2	NSCLC	Non-small cell lung cancer
Cdc7	Protein kinase related to the cell division cycle	OH-HAPs	Hydroxylation of polycyclic aromatic hydrocarbons
CDK6	Cyclin-dependent kinase 6	WHO	World Health Organization
CYP1A1	Cytochrome P450 family 1 subfamily A member 1	P53	Tumor suppressor gene
CYP1B1	Cytochrome P450 family 1 subfamily B member 1	PCK	Phosphoenolpyruvatecarboxykinase
CYP-450	Cytochrome P-450 complex	Pdcd4	Programmed cell death protein 4
CREB	cAMP response element binding protein	PHLPP2	Leucine-rich repeat protein phosphatase
c-Jun	c-Jun N-terminal kinase (JNKs)	PI3K/Akt	Phosphatidylinositol 3-kinase pathway
DBA	Dibenzo(a,h)anthracene	PM_10_	Particulate matter 10
DEP	Diesel engine emission particles	PM_2.5_	Particulate matter 2.5
DEP/PM	Diesel engine emission particles present in particulate matter	PPP2R1B	Protein phosphatase 2 regulatory subunit A, beta
DGCR8	Subunit of the microprocessor complex	WSP	Wood smoke particles
DNA	Deoxyribonucleic acid	PTEN	Homologous tensin phosphatase
DNMT3B	DNA Methyltransferase 3 Beta	RB	Retinoblastoma gene
EGR1	Early growth response protein 1	RBPs	RNA binding proteins
Fl	Fluorene	RISC	RNA-induced silencing complex
Fmo3	Flavin containing monooxygenase	UBQLN	Ubiquilin
GSH	Reduced glutathione	VOCs	Volatile organic compounds
HAEC	Human airway epithelial cell	Wnt/β-catenin	WNT signaling pathway
HAP-DNA	DNA adducts	α4	Alpha 4 protein
PAHs	Polycyclic aromatic hydrocarbons		
HBERT-B[a]P	Human bronchial epithelial cells chemically transformed with B[a]P		
HDAC4	Histone deacetylase 4		
HIF-α1	Hypoxia-inducible factor		
HOXA 1	Homeobox protein A1		
hs-CRP	High sensitivity C-reactive protein		
IARC	International Agency for Cancer Research		
IL-6	Interleukin 6		
